# Construction and validation of cuproptosis-related lncRNA prediction signature for bladder cancer and immune infiltration analysis

**DOI:** 10.18632/aging.204972

**Published:** 2023-08-23

**Authors:** Hanrong Li, Huiming Jiang, Zhicheng Huang, Zhilin Chen, Nanhui Chen

**Affiliations:** 1 Department of Extracorporeal Shock Wave Lithotripsy, Meizhou People’s Hospital (Huangtang Hospital), Meizhou 514031, China; 2 Department of Urology, Meizhou People’s Hospital (Huangtang Hospital), Meizhou 514031, China

**Keywords:** cuproptosis, lncRNA, bladder cancer, prognostic signature, immune environment

## Abstract

Bladder cancer (BC) is a common urologic tumor with a high recurrence rate. Cuproptosis and long noncoding RNAs (lncRNAs) have demonstrated essential roles in the tumorigenesis of many malignancies. Nevertheless, the prognostic value of cuproptosis-related lncRNA (CRLs) in BC is still unclear. The public data used for this study were acquired from the Cancer Genome Atlas database. A comprehensive exploration of the expression profile, mutation, co-expression, and enrichment analyses of cuproptosis-related genes was performed. A total of 466 CRLs were identified using Pearson’s correlation analysis. 16 prognostic CRLs were then retained by univariate Cox regression. Unsupervised clustering divided the patients into two clusters with diverse survival outcomes. The signature consists of 7 CRLs was constructed using the least absolute shrinkage and selection operator (LASSO) Cox regression analyses. Survival curves and receiver operating characteristics showed the prognostic signature possessed good predictive value, which was validated in the testing and entire sets. The reliability and stability of our signature were further confirmed by stratified analysis. Additionally, the signature-based risk score was confirmed as an independent prognostic factor. Gene set enrichment analysis showed molecular alteration in the high-risk group was closely associated with cancer. We then developed the clinical nomogram using independent prognostic indicators. Notably, the infiltration of immune cells and expression of immune checkpoints were higher in the high-risk group, suggesting that they may benefit more from immunotherapy. In summary, the prognostic signature might effectively predict the prognosis and provide new insight into the clinical treatment of BC patients.

## INTRODUCTION

Bladder cancer (BC) is the most common malignant tumor of the urinary system with high morbidity and mortality rates [1]. In 2022, an estimated 81180 new cases and 17100 deaths were expected to occur in the United States alone [2]. Painless hematuria is the most common symptom presented by patients with BC, seen in approximately 80% of cases [3]. According to the depth of tumor invasion, BC can be classified into two major categories, including non-muscle invasive (70-80%) and muscle-invasive BC (20-30%) [4–6]. If diagnosed at an early stage, the 5-year progression-free survival rate of non-muscle-invasive BC is as high as 90 %. However, muscle-invasive BC is characterized by a high incidence of tumor metastasis and a 5-year survival rate ranging from 36% to 48% [7]. Despite the current effective treatment strategies, including surgery, cisplatin-based chemotherapy, and immunotherapy, clinical outcomes are still not satisfactory due to the heterogeneity of its clinical and biologic [8, 9]. The prognosis of BC is much improved by early diagnosis and treatment. However, biomarkers to assist this process are still lacking. To improve clinical diagnosis and treatment of BC, it is imperative to construct an accurate prognostic signature.

Copper is a cofactor for many important enzymes involved in various biological processes and is an essential trace element for nearly all living organisms [10]. Under physiological conditions, intracellular copper concentration must be tightly limited to extraordinarily low levels [11]. Indeed, elevated intracellular copper, even at modest concentrations may be toxic, causing significant cell death [12]. Cuproptosis is a recently recognized form of cell death that is induced by the accumulation of copper in cells, which is distinct from all other known ones [13]. The physiopathological mechanism is that excessive intracellular copper induces the aggregation of lipoylated components of the mitochondrial tricarboxylic acid (TAC) cycle, followed by the loss of iron-sulfur cluster protein, leading to proteotoxic stress and finally cell death [13]. Elevated copper concentrations have been reported to correlate with a wide variety of human cancers [12, 14]. The biological functions of cuproptosis and its impact on the progression of BC are not explicit.

Emerging studies indicated that lncRNAs may serve a significant function in diverse biological functions by regulating gene expression [15, 16]. Dysregulation of lncRNAs contributes to the initiation and development of multiple human cancer and is considered by most to be one of the most specific and sensitive markers [17, 18]. It is confirmed that lncRNA can be utilized to predict outcomes and guide clinical treatments in a variety of cancers [19]. Given the significance of cuproptosis and lncRNAs, some novel methods to provide improved prognostication for BC patients may be a viable strategy.

Here, we systematically investigated the potential roles of cuproptosis-related lncRNA (CRLs) in BC patients using genome sequencing technology and a systematic bioinformatics approach. We constructed a prognostic signature based on 7 CRLs, which could evaluate the prognosis and the effects of immunotherapy in patients with BC. Taken together, the current data in our study may provide a better understanding of the role of CRLs in BC and help in the development of personalized therapy.

## MATERIALS AND METHODS

### Databases

The mRNA expression profiles of 433 bladder cases were obtained from the Cancer Genome Atlas (TCGA, https://portal.gdc.cancer.gov/), which contains 19 normal tissues and 414 bladder tumors. Gene transcription levels were first normalized as fragments per kilobase million (FPKM). The expression level of each transcript was transformed using base log2 (FPKM+1) and low-expression genes with mean expression less than 1 will be filtered out using the “limma” package [20]. Patients with missing survival data and follow-up times less than 30 days were excluded. The related clinical characteristics, copy number variation (CNV), and somatic mutation information with BC were directly acquired from TCGA. According to the human genome annotation (GRCh38), protein-coding genes and lncRNAs were annotated and classified.

### Identification of cuproptosis-related genes (CRGs) and enrichment analysis

Seventeen cuproptosis-related genes (CRGs) were sourced from a recent article (2022) appearing in Cell [13]. The differential expression analysis for CRGs between the tumor and normal tissues was completed with R package “limma” using the thresholds of |log2FC| > 1 and P <0.05. The R package “maftools” was used for the aggregation and visualization of the mutation landscape [21]. The correlations of CPGs were created using the R package “igraph”. GeneMANIA (http://genemania.org/) was utilized for constructing a weighted functional interaction network, which is a web tool that can predict gene interactions and screen other potential binding partners [22]. The Gene ontology (GO) biological meaning and Kyoto Encyclopedia of Genes and Genomes (KEGG) pathway enrichment of the CRGs were performed to uncover the function through the R package “clusterProfiler” [23]. The corrected P < 0.05 was considered significantly enriched.

### Identification of cuproptosis-related lncRNAs (CRLs) and consensus clustering analysis

Cuproptosis-Related lncRNAs (CRLs) were identified by using the Pearson correlation index calculation between CRGs and lncRNAs. On the premise of correlation coefficient > 0.05 and P < 0.05, certain lncRNAs can be regarded as CRLs. Univariate Cox regression analysis was conducted to identify the prognostic-associated CRLs (P < 0.01). The prognostic CRLs were then used for unsupervised clustering and classification with the R package “consensusClusterPlus” [24]. To produce the most stable consensus, we repeated the optimization procedure 50 times with 80% item resampling. Kaplan-Meier analysis was used to assess the differences in overall survival (OS) between different subgroups. In addition, the comparison of clinicopathological factors and the expression of PD-L1 in different clusters was performed.

### Construction and validation of CRL prognostic signature

The included cases (n = 403) were split in a 1:1 to ratio training (n=203) and testing (n=200) sets. We then executed the least absolute shrinkage and selection operator (LASSO) on the above prognostic CRLs to construct the signature in the training set. The risk score of each patient was generated by the sum of multiplying the expression value of each CRL by its regression coefficient. According to the optimal cutoff value, patients were classified into low- and high-risk groups. Prognostic differences between the two groups were revealed using Kaplan-Meier survival curves, and receiver operating characteristic (ROC) curves were plotted for 5 years to measure the predictive ability of this signature. Meanwhile, we applied the testing and entire sets to validate the above findings.

### Stratification survival analyses and clinical significance

To investigate the clinical value of the prognostic signature, patients were stratified based on different clinical variables. In the stratified analysis, prognostic differences in different groups were analyzed by means of Kaplan-Meier and log-rank test survival analysis. We then applied the R package “pheatmap” to plot a heatmap to illustrate the distribution of clinical characteristics in diverse groups using the Chi-square test.

### Gene set enrichment analysis

Gene set enrichment analysis (GSEA) (https://www.gsea-msigdb.org/gsea/login.jsp) software was used to investigate potential mechanisms [25]. KEGG and HALLMARK gene sets were used as references. The adjusted P value (false discovery rate) < 0.25 and normalized enrichment score > 1 denoted statistical significance.

### Nomogram construction

The nomogram can predict the probability of a certain clinical outcome based on the values of multiple variables [26]. We conducted univariate and multivariate Cox regression analyses to assess the independent prognostic value of prognostic signature and clinical parameters (age, gender, grade, and stage). Then, variables that could be used as independent prognostic indicators were selected to develop a nomogram in BC using the R package “RMS” package. The predictive performance of the nomogram was then validated by calibration curves and decision curve analysis (DCA).

### Tumor mutation burden and drug sensitivity analysis

Tumor Mutational Burden (TMB) is a somatic biomarker proposed to predict response to immunotherapies in cancer [27]. TMB was counted and visualized in each sample using the R package “maftools” [21]. In addition, we compared TMB between two different groups and plotted the risk score survival curve for TMB. Moreover, half maximal inhibitory concentration (IC50) values of different drugs in the low- and high-risk groups of bladder cancer samples were calculated with the R package “oncoPredict” [28].

### Exploration of immune features and prediction of immunotherapy efficacy

To explore the relationship between prognostic signature and immunity, the infiltration status and immune function were analyzed between two groups. Multiple immune data platforms, including TIMER, CIBERSORT, CIBERSORT-ABS, QUANTISEQ, MCPCOUNTER, XCELL, and EPIC algorithms were used to estimate the immune infiltration status of patients with bladder cancer. The single sample gene set enrichment analysis (ssGSEA) was used to calculate the enrichment score of 29 immune cells, functions, or pathways [29]. The tumor microenvironment score of each sample was calculated using the R package “Estimate”. Furthermore, the expression levels of several common immune targets were compared between the two groups. The Tumor Immune Dysfunction and Exclusion (TIDE) database (http://tide.dfci.harvard.edu/) [30] was used to further analyze the difference of TIDE score in the low- and high-risk groups of BC samples.

### Statistical analysis

All statistical analyses were performed in R statistical software (version 4.0.1) and Perl language (version 5.30.2). Unless otherwise specified, statistical significance was considered for two-tailed P < 0.05.

## RESULTS

### The expression profiles and transcriptional alterations of CRGs

**Figure 1:**
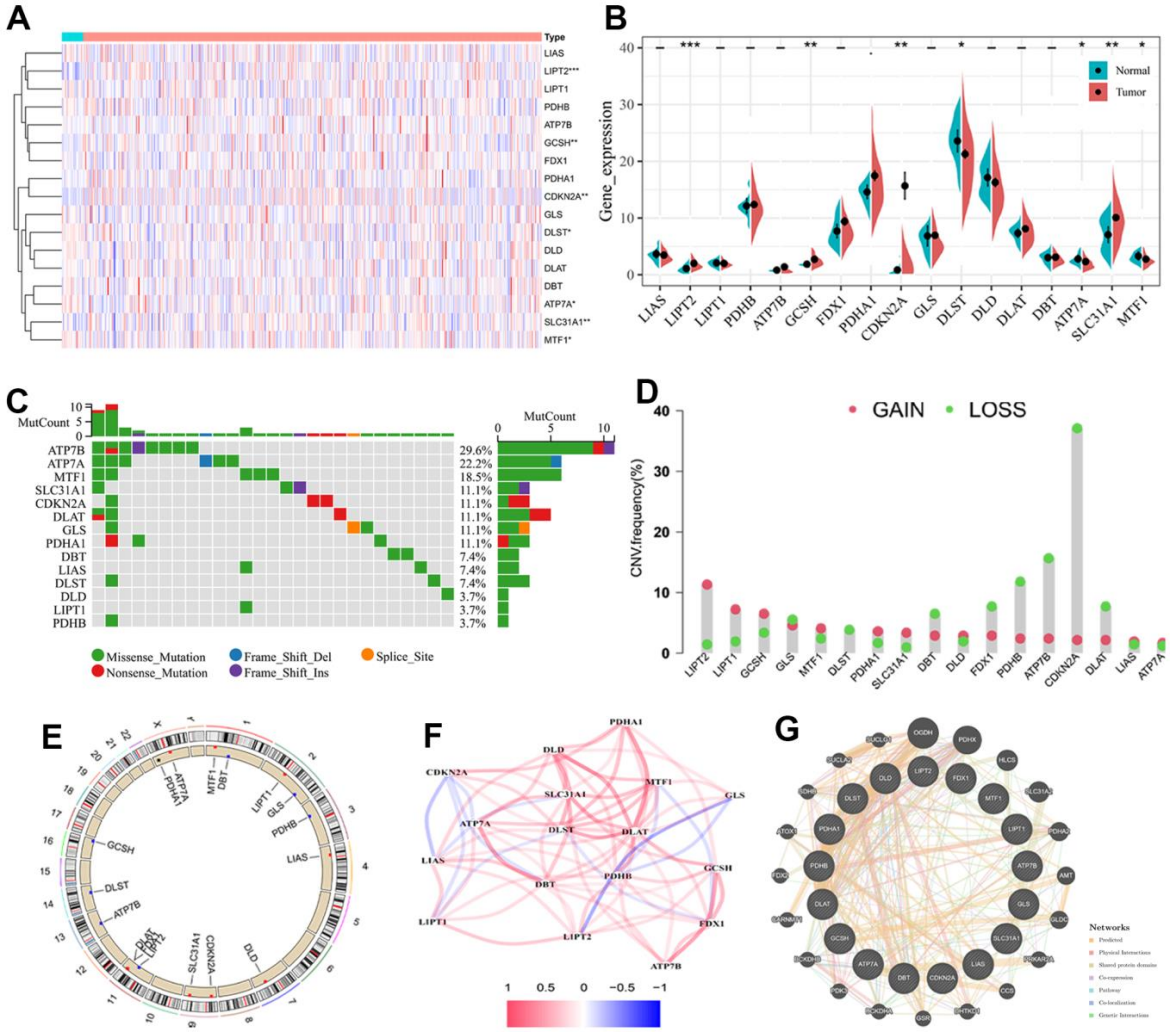
**The multi-omics landscape of the cuproptosis-related genes (CRGs) in BC.** (**A**) Heatmap of the CRGs in BC and normal samples. (**B**) Violin plot showing the differences in the expression of 17 CRGs between BC and normal tissues. (**C**) Genetic alterations of CRGs in BC. (**D**) CNV mutation frequency of CRGs in BC. (**E**) Location of CNV alterations in BC. (**F**) The relationship network of the CRLs. Red and Blue lines indicate positive and negative correlations, respectively. (**G**) The regulatory network of 17 CRGs and additional 20 genes spontaneously pulled through GeneMANIA. -, no significant, * P < 0.05, ** P < 0.01.

To explore the function of cuproptosis in BC, the expression profiles of 17 CRGs in BC samples were compared with that of normal samples. In total, seven CRGs were identified as differentially expressed genes. Compared with normal bladder tissues, four genes (LIPT2, GCSH, CDKN2A, and SLC31A1) were determined to be up-regulated, while three genes (DLST, ATP7A, and MTF1) were down-regulated in BC cases (Figure 1A, 1B). We then examined the alteration frequency and CNVs in 17 CRGs. As can be seen from Figure 1C, all genes went through some degree of mutations and ATP7B had the highest frequency of mutations. Subsequently, we explored the CNVs duplicated or deleted in the segments of the genome and noticed the alterations were common among CRGs. CDKN2A had the highest amplification, while LIPT2 showed an extensive deletion in CNV (Figure 1D). The CNV chromosome location information of 17 CRGs was displayed in Figure 1E. The results suggested that these CRGs were scattered in different chromosomes. The relationship network revealed a highly intricate interaction network of these CRGs (Figure 1F). Moreover, the regulatory network consisted of 37 genes, including 17 CRGs and additional genes spontaneously pulled through GeneMANIA. The biological functions association and relevant gene networks were visualized in Figure 1G.

### Biological function of CRGs

**Figure 2:**
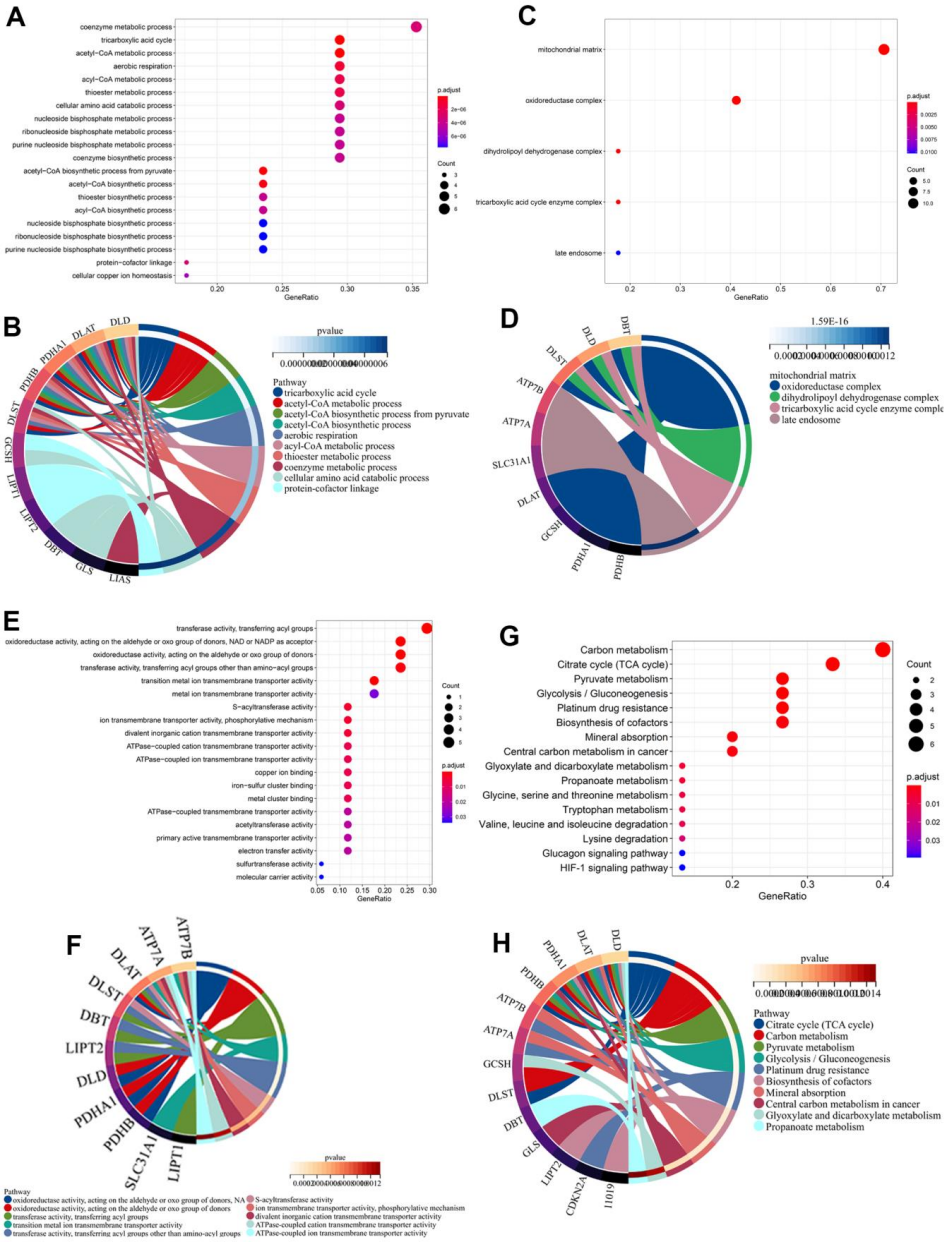
**Functional enrichment analysis of cuproptosis-related genes (CRGs) in BC.** The bar plot and circle plot of enriched GO BP (**A**, **B**), CC (**C**, **D**), MF (**E**, **F**), and KEGG (**G**, **H**) of CRGs in BC.

GO analysis showed these 37 genes (including 17 CRGs and additional 20 genes spontaneously pulled from GeneMANIA) were enriched in the coenzyme metabolic process, tricarboxylic acid cycle, and acetyl-CoA metabolic process in biological processes (BP) (Figure 2A, 2B). The top three significantly enriched cellular component (CC) were mitochondrial matrix, oxidoreductase complex, and dihydrolipoyl dehydrogenase complex (Figure 2C, 2D). As for molecular function (MF), oxidoreductase activity and transferase activity-transferring acyl groups were most significantly enriched (Figure 2E, 2F). In parallel, KEGG analysis revealed these genes were involved in carbon metabolism, citrate cycle, and pyruvate metabolism (Figure 2G, 2H).

### Identification of CRLs and construction of co-expression network

**Figure 3:**
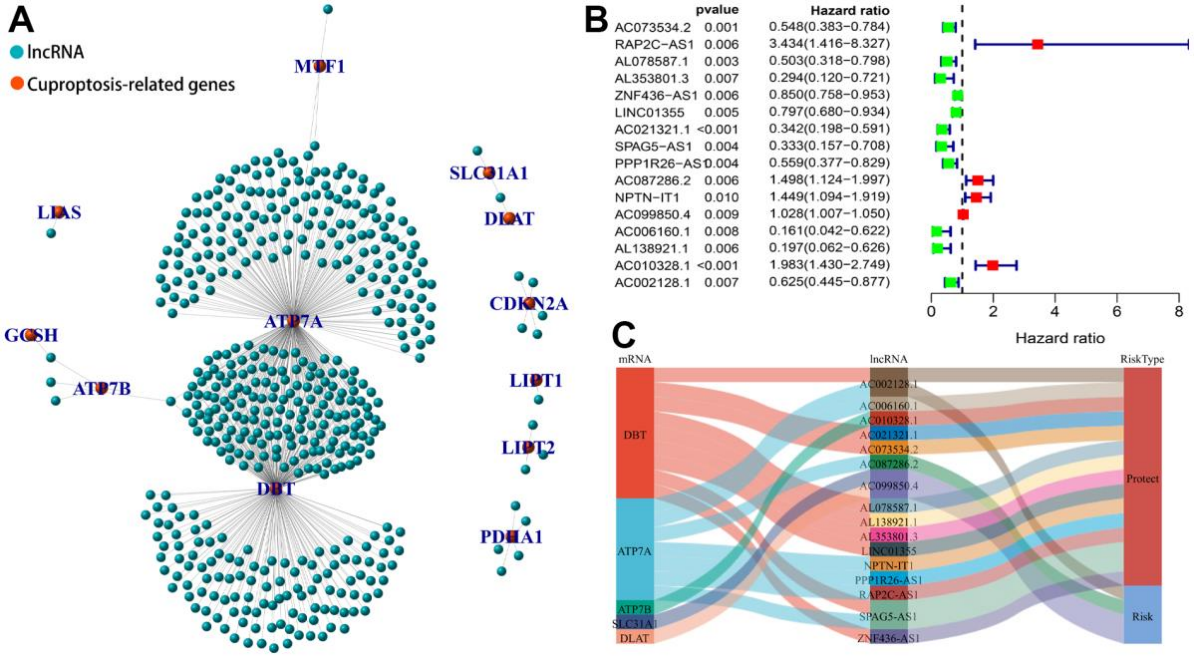
**Identification of cuproptosis-related lncRNA (CRLs) in BC.** (**A**) The co-expressed network between 17 cuproptosis-related genes and 466 lncRNAs. (**B**) The forest plot shows 16 prognostic CRLs in univariate Cox regression analysis. (**C**) Sankey graph of the co-expression network.

We performed a Pearson’s correlation analysis between all lncRNAs and 17 CRGs based on both expression profiles in BC tissues. A total of 466 CRLs were screened out with the filtering criteria. The results of the co-expression network between 12 CRGs and 466 lncRNAs were presented in Figure 3A. We then conducted a univariate Cox regression analysis on these screened CRLs and discovered that 16 CRLs were significantly related to OS (P< 0.01), including 5 risk (HR > 1) and 11 protective (HR < 1) lncRNAs in BC patients (Figure 3B). The Sankey diagram presented the correspondence between CRGs and CRLs (Figure 3C).

### Determine the cuproptosis subtypes based on the prognostic CRLs

**Figure 4:**
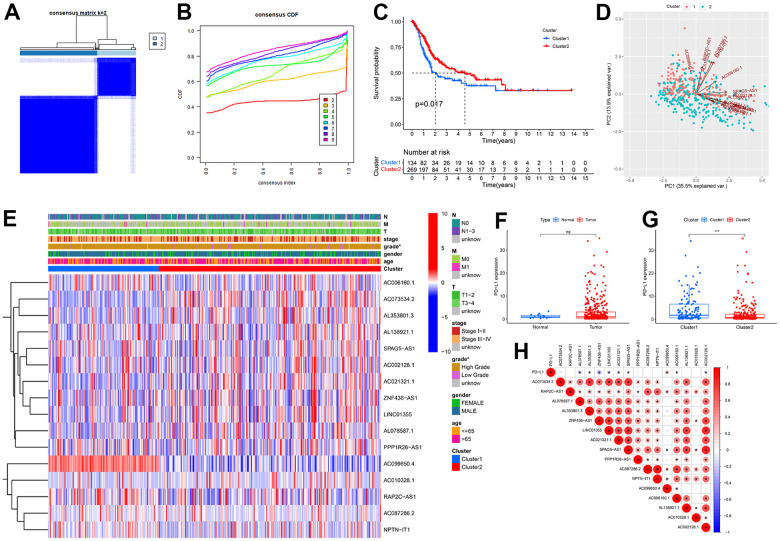
**Unsupervised consensus clustering of 16 prognostic cuproptosis-related lncRNA (CRLs) in BC.** (**A**) Consensus matrix that divides BC cases into two clusters (k=2). (**B**) Cumulative distribution function (CDF) curves for k = 2–9. (**C**) Kaplan Meier analysis for BC patients in two clusters. (**D**) PCA showed the difference in distribution between the two clusters. (**E**) The distribution of clinical features and expression of 16 CRLs between two clusters. (**F**) No differential expression was observed for PD-L1 between BC and normal tissues. (**G**) PD-L1 upregulation in cluster1. (**H**) The correlation of PD-L1 with CRLs. * P<0.05, ** P<0.01, ns: no significant.

To better understand the molecular heterogeneity of BC based on the 16 prognostic CRLs, unsupervised consensus clustering was conducted to group BC samples. According to the consensus matrixes (Figure 4A) and cumulative distribution function (CDF) curves (Figure 4B), k = 2 was the most optimal value to divide the BC cases into two subtypes (cluster1, n = 134; cluster2, n = 269). Kaplan-Meier analysis demonstrated a considerable difference in OS between two clusters, with cluster2 having a significant survival advantage (Figure 4C). The PCA reconfirmed the difference in distribution between the two clusters (Figure 4D). The distribution of clinical variables and expression of 16 CRLs between two clusters were intuitively shown in a heatmap (Figure 4E). Patients in cluster2 were more related to the high-grade relative to cluster1.

To examine the involvement of PD-L1 with CRLs, we analyzed the expression of PD-L1 between two subtypes. It was observed that expression levels of PD-L1 in BC tissues were not significantly different from that in normal tissues (Figure 4G). Notably, cluster1 had a statistically distinctly higher PD-L1 level than cluster2 (P < 0.001) (Figure 4G). Next, we plot a heatmap to study the correlation between the PD-L1 and 16 CRLs and found weak but significant negative relations between the CRGs and most CRLs (Figure 4H).

### Construction and validation of the prognostic signature

**Figure 5:**
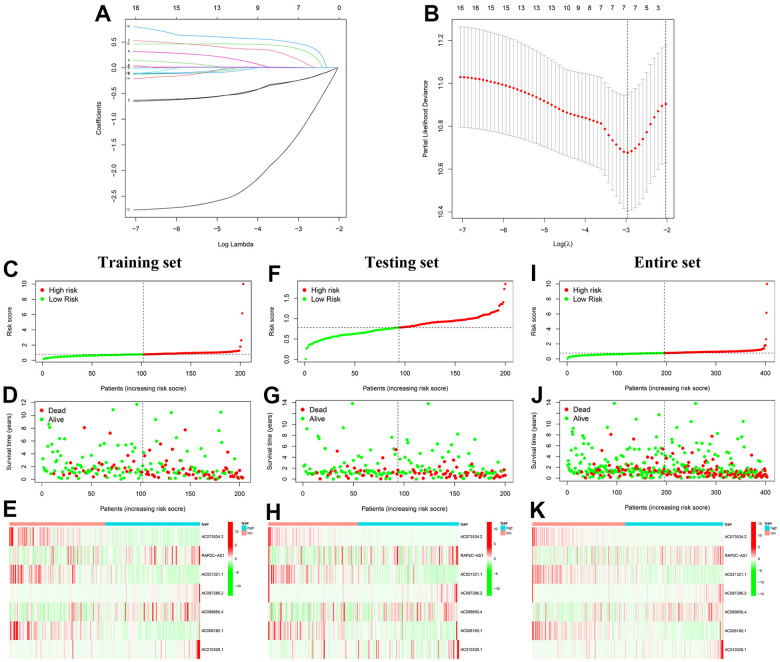
**Construction and validation of the prognostic signature.** (**A**) The 10-fold cross-validation for the optimal parameter selection in the LASSO regression. (**B**) The profile of the LASSO coefficient. (**C**) The distribution plots of the risk score in the training set. (**D**) The survival status of BC patients in the training set. (**E**) The heatmap of 7 CRLs in the training set. (**F**) The distribution plots of the risk score in the testing set. (**G**) The survival status of BC patients in the testing set. (**H**) The heatmap of 7 CRLs in the testing set. (**I**) The distribution plots of the risk score in the entire set. (**J**) The survival status of BC patients in the entire set. (**K**) The heatmap of 7 CRLs in the entire set.

In the training set, we performed LASSO Cox regression analysis on 16 prognostic CRLs to establish a prognostic signature. The optimal signature was constructed using 7 CRLs when the log (lambda) was the least deviation possibility (Figure 5A, 5B). We calculated the risk score of each patient and the formula was generated as follow: risk score = (-0.236 × expression of AC073534.2) + (0.141 × expression of RAP2C-AS1) + (-0.238 × expression of AC021321.1) + (0.463 × expression of AC087286.2) + (0.005 × expression of AC099850.4) + (-1.239 × expression of AC006160.1) + (0.358 × expression of AC010328.1). In the training set, patients were separated into a low-risk group (n = 102) or a high-risk group (n = 101) with the optimal cutoff value (Figure 5C). Moreover, as the risk score increased, so did the mortality rate (Figure 5D). Heatmap representing the expression levels of the 4 risk lncRNAs (RAP2C-AS1, AC087286.2, AC099850.4, and AC010328.1) were much higher in the high-risk groups relative to controls. As expected, the other 3 protective lncRNAs (AC073534.2, AC021321.1, and AC006160.1) showed opposite expression trends (Figure 5E). Similar findings can be also found using the same method on the testing set (Figure 5F–5H) and the entire set (Figure 5I–5K).

**Figure 6:**
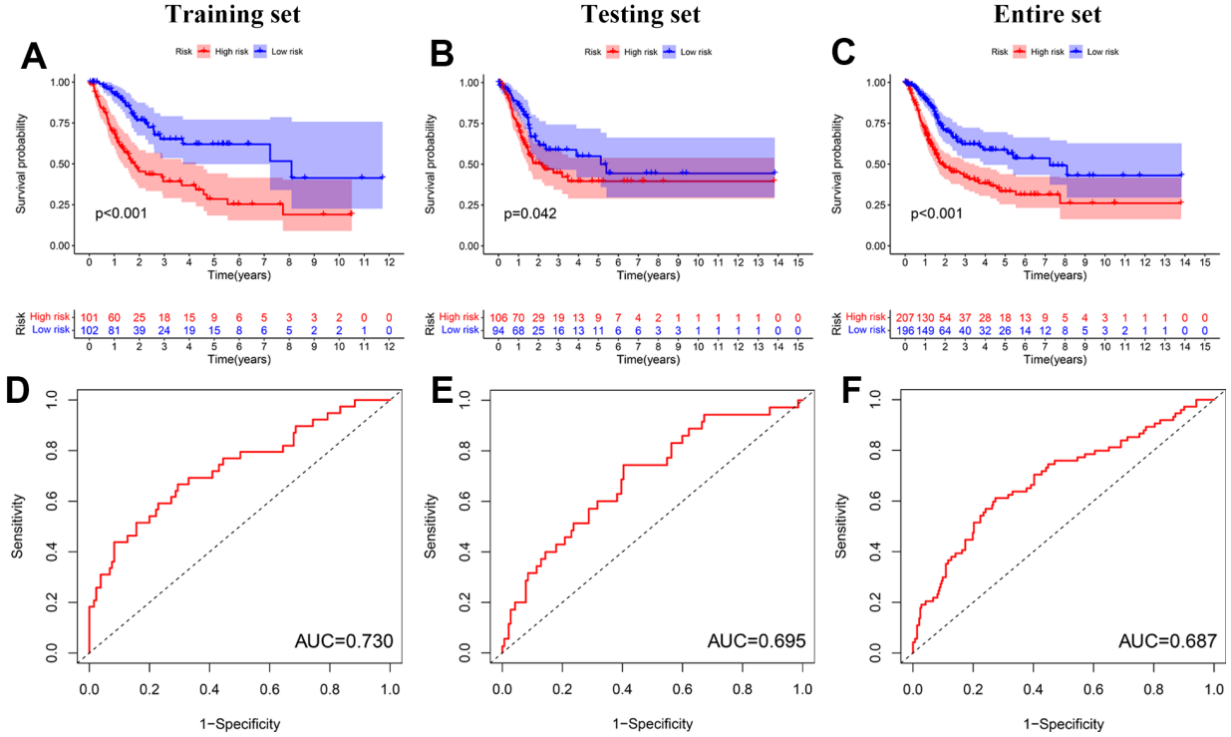
**Prognosis value of the prognostic signature.** (**A**–**C**) Kaplan–Meier survival curves of BC patients in the training, testing, and entire sets. (**D**–**F**) ROC analyses in the training, testing, and entire sets.

To compare prognosis between two groups, the Kaplan-Meier curve was performed with the log-rank test. The OS of the low-risk group was remarkably longer than that of the high-risk group in the training (P < 0.001), the testing (P = 0.042), and the entire sets (P < 0.001) (Figure 6A–6C). Furthermore, we constructed the ROC curves and calculated the area under the ROC curve (AUC) to evaluate the accuracy of diagnoses. The AUC values of the signature for predicting 5-year survival rates were 0.730, 0.695, and 0.687 in the training, the testing, and the entire sets, respectively (Figure 6D–6F).

### Clinical application of the prognostic signature

Stratification survival analyses were employed to evaluate the prediction power of the constructed signature in diverse subgroups of clinical parameters. As shown in Figurer 7, patients in the high-risk group suffered a poorer outcome than patients in the high-risk group for all subgroups other than cases with low-grade, or with T1-2 stage, or with N1-3 stage, or with M1 stage. These results indicated this signature had good predictive power.

**Figure 7:**
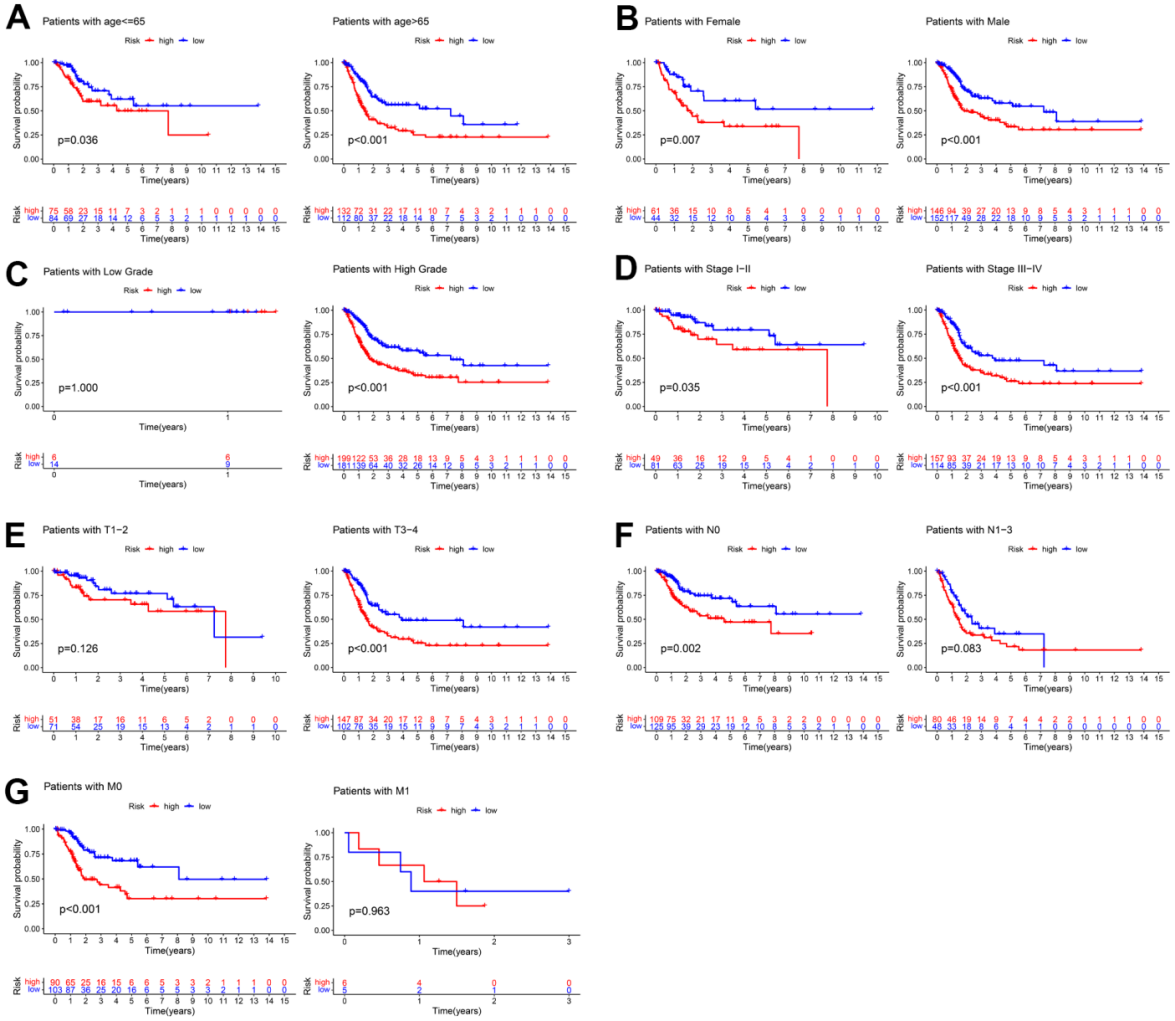
**Kaplan–Meier survival curves of the low- and high-risk groups in different subgroups of clinical parameters.** (**A**) age, (**B**) gender, (**C**) grade, (**D**) AJCC stage, (**E**) T stage, (**F**) N stage, (**G**) M stage.

**Figure 8:**
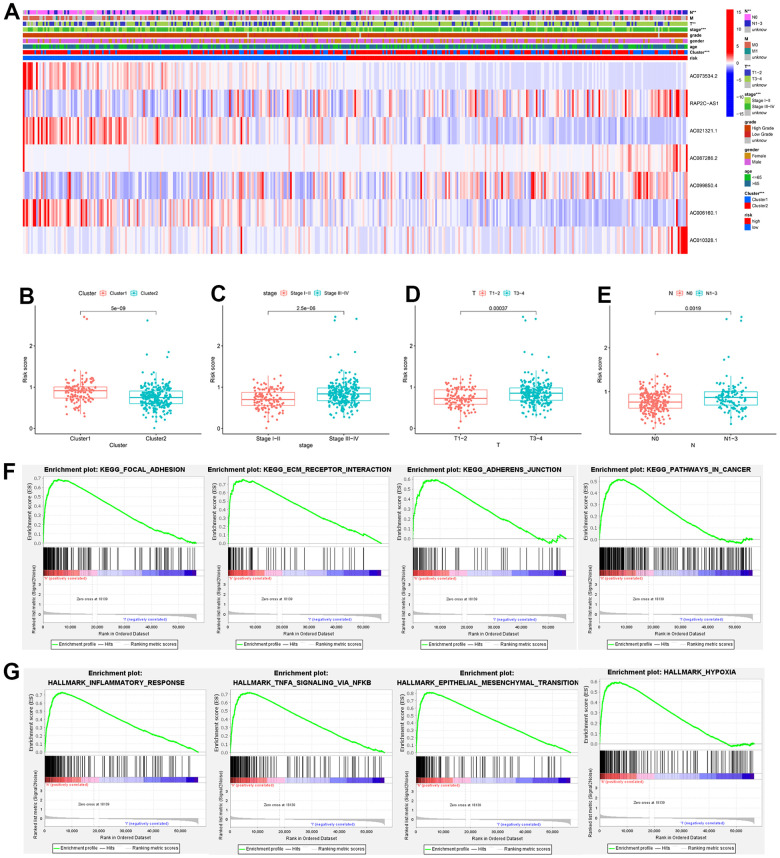
**Heatmap of the clinical relevance and GSEA.** (**A**) Heatmap of the distribution of clinical features and expression of 7 CRLs between two groups. (**B**–**E**) Differential expression analysis of risk score in patients with different clusters, AJCC stage, T stage, and N stage. (**F**) GSEA showed the significantly enriched KEGG gene sets in the high-risk group. (**G**) GSEA showed the significantly enriched Hallmark gene sets in the high-risk score. ** P<0.01,*** P<0.001.

The association between clinical features and risk score was further investigated. Significant differences were observed for various clinical features in terms of cluster (P < 0.001), AJCC stage (P < 0.001), T stage (P < 0.01), and N stage (P < 0.01) between the high- and low-risk groups (Figure 8A). We also explored the relationship between risk score, and subtype, AJCC stage, T stage and N stage. Patients in cluster1 had significantly higher risk scores than those in cluster2 (Figure 8B). In addition, the risk score increased along with the AJCC stage, T stage, and N stage increased (Figure 8C–8E).

To further explore the potential biological functions and pathways between low- and high-risk groups, GSEA was conducted. As anticipated, the cancer-related pathways were obviously associated with the high-risk group, such as focal adhesion (Figure 8F) and epithelial-mesenchymal transition (Figure 8G).

### Development of the nomograph

**Figure 9:**
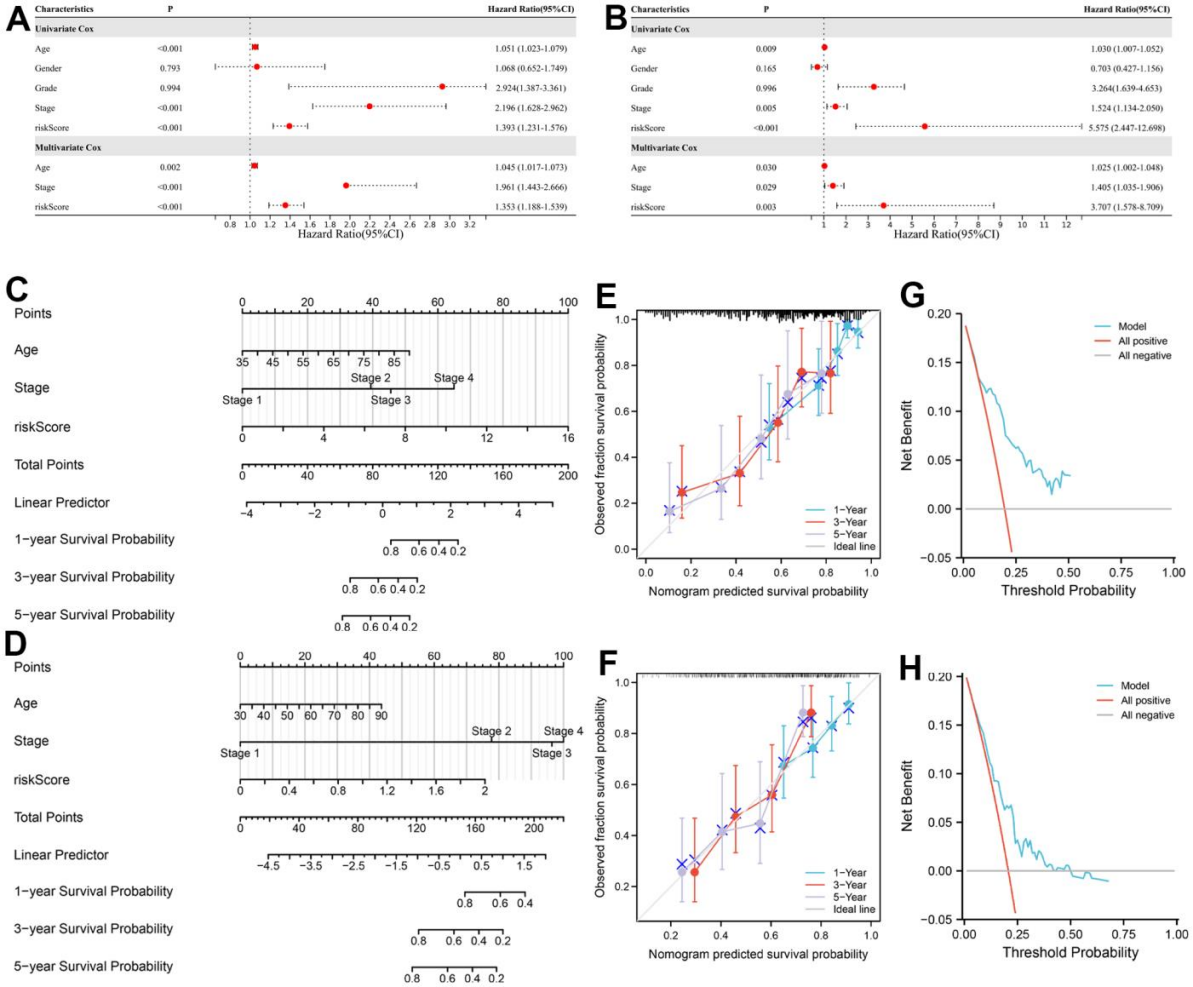
**Development of a nomogram for predicting the prognosis of BC patients.** (**A**, **B**) Uni- and multi-variate Cox regression analysis of the clinical parameters in the training and testing sets. (**C**, **D**) Nomogram for predicting the 1-, 3-, and 5-year OS of BC patients in the training and testing sets. (**E**, **F**) Calibration curves of nomogram in the training and testing sets. (**G**, **H**) DCA of nomogram in the training and testing sets.

Subsequently, we conducted Cox regression analyses to determine the independent prognostic parameters for BC patients. In the training set, the univariate Cox analysis indicated age (P < 0.001), AJCC stage (P < 0.001), and risk score (P < 0.001) were significantly associated with the OS. Further multivariate Cox analysis suggested that age, AJCC stage and risk score remained significant (Figure 9A). A similar result was acquired with the same technique from the testing set (Figure 9B). The above results demonstrated that prognostic signature was an independent prognostic predictor for OS in patients with BC.

Based on the Cox regression analyses, the clinical nomogram was developed using three independent prognostic factors as parameters in the training (Figure 9C) and the testing sets (Figure 9D). Each factor was assigned a score in proportion to its risk contribution to survival, and the total score could be accordingly calculated. The calibration chart of the nomogram showed fairly consistent in predicting OS of the nomogram with the observing 1-, 3- and 5-year results in both sets (Figure 9E, 9F). In addition, the result of DCA demonstrated this clinical nomogram owned good net benefits in predicting the prognosis in both sets (Figure 9G, 9H).

### TMB and drug analysis

**Figure 10:**
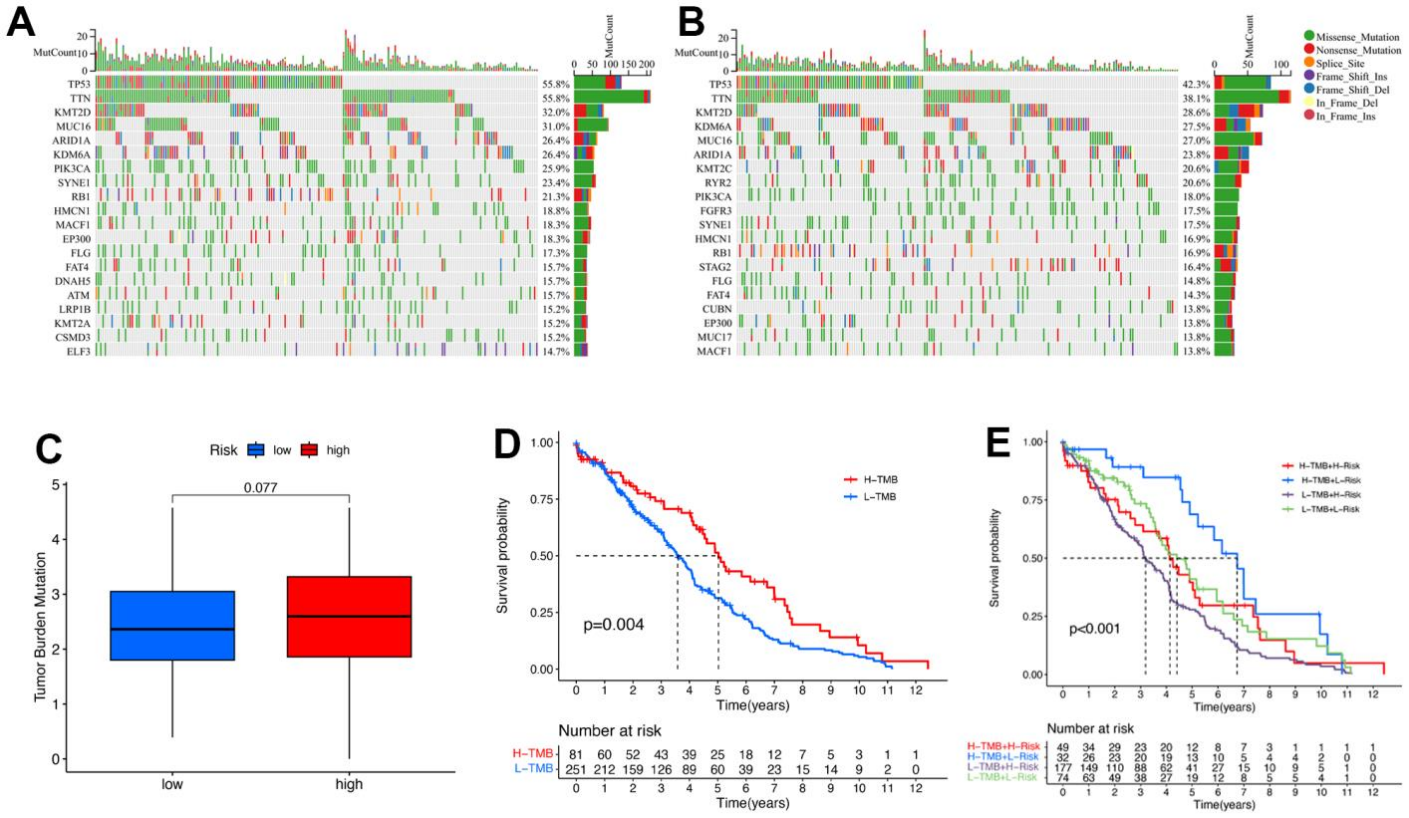
**The relationship between the signature and TMB.** Top 20 mutation genes of BC for the low-risk (**A**) and high-risk (**B**) groups in waterfall plot. (**C**) TMB comparison between low- and high-risk groups. (**D**) Kaplan-Meier curves for high- and low-TMB groups. (**E**) Kaplan-Meier curves for the patients stratified by TMB and risk scores.

We also explored the differences in TMB levels between the low- and high-risk groups. We found that the 5 most highly mutated genes were TP53, TTN, KMT2D, MUC16, and ARID1A in the high-risk group, which TP53, TTN, KMT2D, KDM6A, MUC16, and ARID1A in the low-risk group (Figure 10A, 10B). This difference was not statistically significant (P = 0.077), although considerably high levels of TMB were observed in the high-risk group (Figure 10C). Importantly, we noticed a remarkable difference in the survival analysis of TMB between the low- and high-risk groups (Figure 10D). High TMB had a longer survival time than low TMB (P = 0.004). Meanwhile, there was a statistically significant difference in the combined analysis of TMB and patient risk (Figure 10E, P < 0.001).

**Figure 11:**
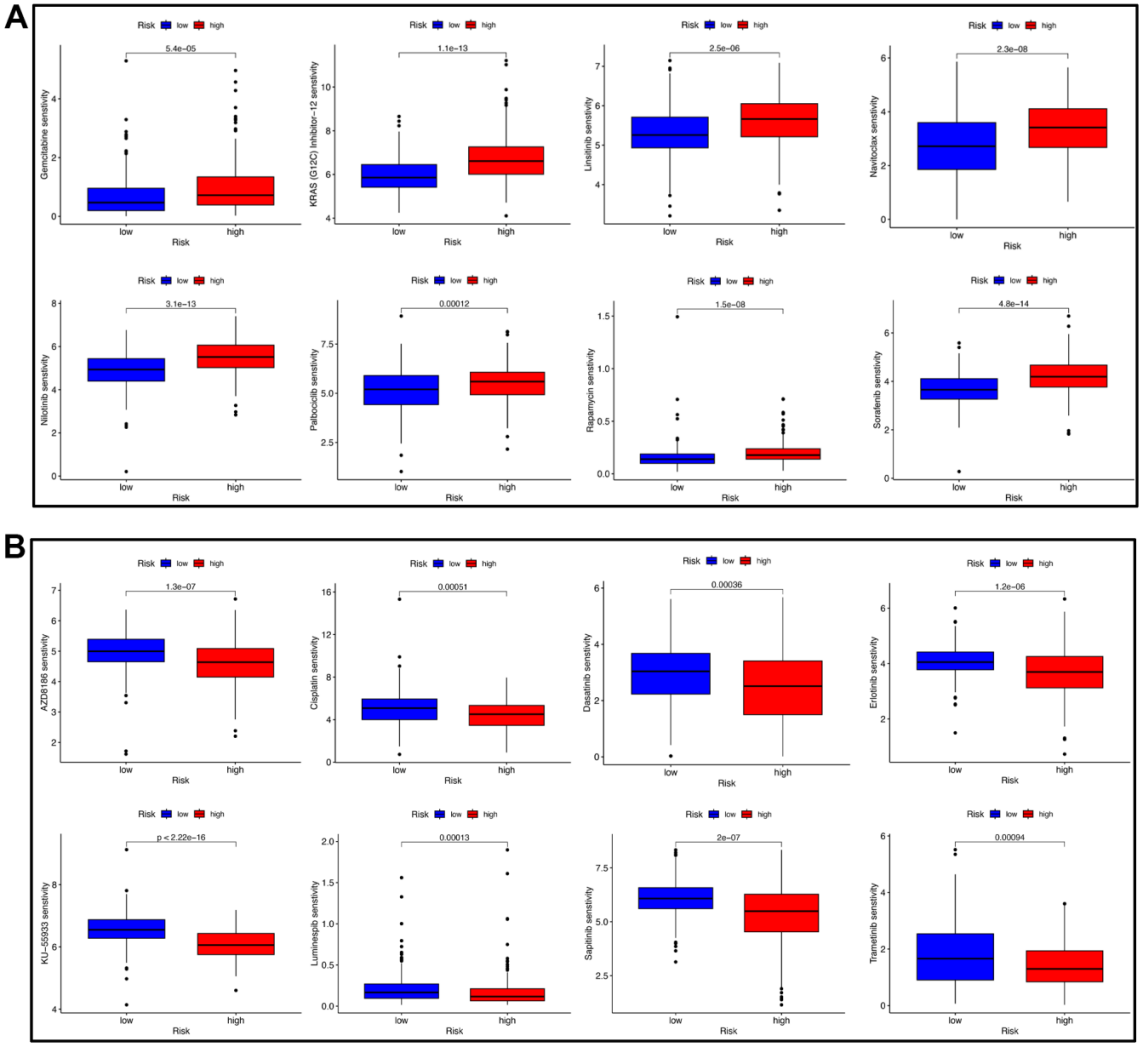
**Drug sensitivity analysis.** (**A**) BC patients with a high-risk score had a higher IC_50_ value of eight therapeutic drugs compared with patients with a low-risk score. (**B**) BC patients with a high-risk score had a lower IC_50_ value of many therapeutic drugs compared with patients with a low-risk score.

Subsequently, potential anti-tumor drugs were screened through the algorithm from the “oncoPredict” R package. We calculated the IC_50_ of common agents in the low- and high-risk groups and found that patients in the high-risk group were significantly more sensitive to gemcitabine, KRAS (G12C) inhibitor-12, linsitinib, navitoclax, nilotinib, palbociclib, rapamycin, sorafenib, and temsirolimus (Figure 11A), while AZD8186, cisplatin, dasatinib, erlotinib, KU-55933, luminespib, sapitinib, and trametinib had higher IC_50_ values in the low-risk group (Figure 11B). These findings showed that low- and high-risk group had the corresponding drug susceptibility patterns, suggesting that the risk score might distinguish more suitable patients to receive appropriate chemotherapies.

### Exploration of immunological features and immunotherapy response

**Figure 12:**
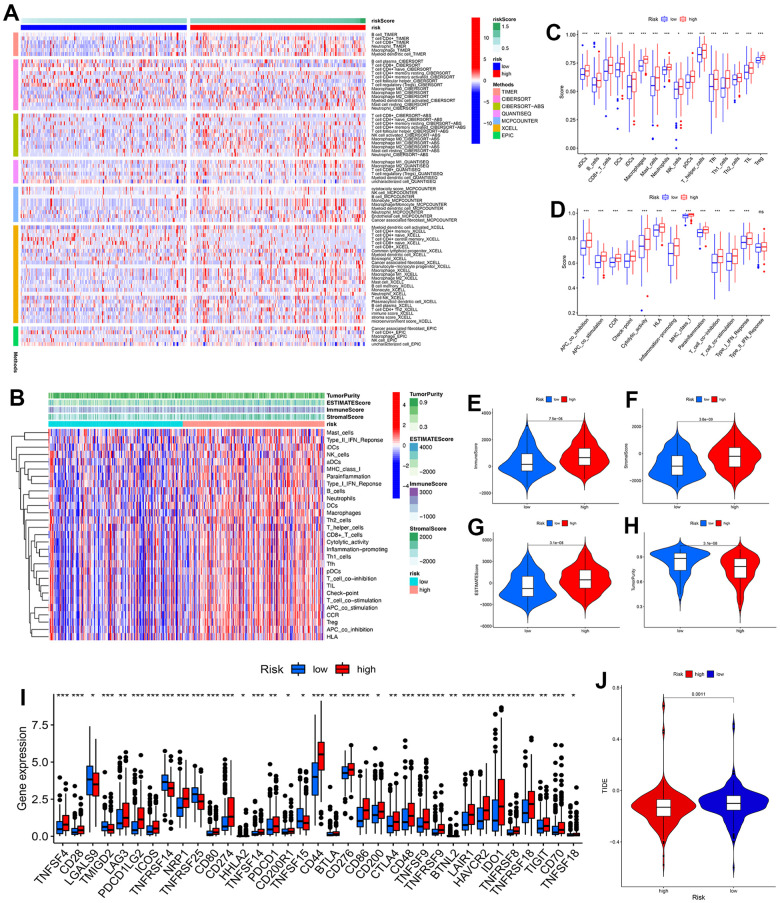
**Analyses of immunological features and immunotherapy response.** (**A**) Heatmap showed the expression of immune cells between low- and high-risk groups. (**B**) Enrichment levels of immune-related cells and types between the low- and high-risk groups in the heatmap. (**C**) Differences in immune cells between two groups. (**D**) Differences in immune function between two groups. (**E**–**H**) Comparison of immune score, stromal score, ESTIMATE score, and tumor purity between two groups. (**I**) Differences in immune checkpoint expression between two groups. (**J**) TIDE comparison between low- and high-risk groups. * P<0.05, ** P<0.01, *** P<0.001.

To investigate the profile of immune infiltration in BC, the relationship between risk score and immune cell infiltration was estimated using 7 algorithms. The heatmap showed that a considerable number of immune cells had elevated expression in the high-risk group than in the low-risk group (Figure 12A) We quantified the enrichment level of 13 immune cells and 16 immune functions for each patient using a ssGSEA algorithm (Figure 12B–12D). Significant differences in all 29 immune infiltrating signatures were found between the two groups, and the higher expression occurred in the high-risk group. Consistently, the high-risk group also represented a higher immune score, stromal score, and ESTIMATE score and lower tumor purity (Figure 12E–12H). Taken together, these results suggested the prognostic signature might be related to the tumor immune microenvironment. Given the important clinical advances of immune checkpoint inhibitor (ICI) therapy in various tumors, an assessment was done to evaluate the distribution of 47 ICI–relevant genes between two groups. The expression levels of many ICIs were markedly up-regulated in the high-risk group than those in the low-risk group (Figure 12I). In addition, BC patients with a low-risk score had a higher TIDE score than those with a high-risk score (Figure 12J, P = 0.0011). A higher TIDE score represents higher possibility of immune escape, indicating less benefit from immunotherapy. Taken together, these findings revealed that ICI therapy might be a potentially effective therapeutic modality for the high-risk group.

## DISCUSSION

BC is one of the most common cancers worldwide, whose morbidity and mortality are increasing in recent years [5]. The issues of tumor recurrence, malignant behavior, and drug resistance continue to be a challenge in the therapeutic and prognostic management of BC [31]. There is considerable urgency to construct precise predictive methods to promote the prognosis and treatment of BC.

A recent study reported that in addition to directly targeted cytotoxicity caused by the dysregulation of copper homeostasis, alteration in intracellular copper levels may contribute to cancer initiation and progression [32]. Cuproptosis is an emerging programming form of cell death that differs from other known death modes, which has been proven to be involved in mitochondrial respiration and the TCA cycle [13]. Growing evidence supports this notion that dysregulated copper homeostasis could influence tumor growth and progression [12], and copper exhibits an essential characteristic in tumor immune response and antitumor therapy [33, 34]. LncRNA expression is the most pervasive transcriptional alteration in cancer to a recent survey of transcriptome studies of human cancers [35]. LncRNAs have been found to serve significant roles in tumorigenesis [17]. However, until now little is known about the role of CRLs in BC.

To our knowledge, no previous data on the correlation between CRLs and biological and clinical features in BC have been reported. In this study, we first described the expression pattern, mutation landscape, and functional annotation of CRGs in BC. In agreement with prior studies, most of the CRGs were altered in BC, including ATP7B [36]. Meanwhile, the functional annotation revealed that these genes were significantly associated with coenzyme metabolic process, mitochondrial matrix, citrate cycle (TCA cycle), and so on [13, 37, 38].

We then identified 466 CRLs according to the co-expression analysis. 17 CRLs related to the prognosis of BC patients were retained for subsequent analysis. Two distinct subtypes, that is, cluster1 and cluster2, were determined based on 7 prognostic CRLs in BC via consensus clustering. The cluster subtype affected the prognosis and tumor grade and was associated with PD-L1. Among them, cluster1 exhibited a poorer prognosis and higher expression of PD-L1, suggesting those patients acquire much more benefit.

Increasingly, researchers have developed multiple-marker models to assess outcomes of patients with tumors. Meanwhile, the predictive signature based on CRLs has become a hotspot for recent research. Mo et al. [39] developed a signature that may serve as a marker for prognosis prediction for lung cancer, and the cuproptosis-related ceRNA regulatory axis might contribute to gene therapy. Xu et al. [40] have developed a signature containing 10 CRLs to help evaluate the prognosis and molecular profile of clear cell renal cell carcinoma. However, the prognostic value of CRLs in BC remains to be studied.

To evaluate the outcome more accurately and promote treatment decisions for BC patients, we constructed the prognostic signatures based on 7 CRLs using LASSO regression analysis. Among them, there is evidence that the expression pattern of AC073534.2 may indicate its role in acute myeloid leukemia and was associated with a favorable prognosis [41]. RAP2C-AS1 has been reported to be highly expressed in esophageal cancer and associated with an unfavorable prognosis [42]. A study revealed that AC087286.2 was determined as a risk factor with HR > 1 for gastric cancer [43]. In addition, AC099850.4 has also been reported in hepatocellular carcinoma and high-grade ovarian cancer [44, 45]. Liu et al. found AC006160.1 overexpression inhibited BC cell proliferation and invasive abilities, serving as a protective lncRNA for the progression of BC [46]. However, there were few reported cases in the literature regarding AC021321.1 and AC010328.1.

Not only did we obtain the most accurate model, but also calculate the optimal cutoff values to distinguish the low- or high-risk group among BC patients. We further demonstrated a significant difference in survival time between the low-and high-risk groups. Obviously, patients in the high-risk group had a significantly poor prognosis. The ROC curves validated the performance of the prognostic signature. The stability and effectiveness of the signature were verified using stratification survival analyses. Surprisingly, we noticed that the risk score was closely linked with the cluster subtype. The risk score significantly varied between BC patients with different AJCC, T, and N stages, revealing risk score was positively linked with tumor progression. More specifically, the risk score increased as the disease progressed. GSEA results revealed the cancer-related pathways were highly associated with the high-risk group, implying that cuproptosis participated in the development of BC.

The results of the current study also demonstrated that signature-based risk score as well as age and AJCC stage were independent prognostic parameters for BC patients. Nomograms are effective tools for predicting tumor prognosis via a simple visualization format [47]. In clinical practice, an accurate prognostic nomogram can contribute physicians to make clinical reliable decisions or guide adjuvant therapy, especially in vulnerable patients with a high risk of death [48]. Next, we developed a nomogram using the various independent clinical factors (including risk score, age, and stage) to calculate the probability of OS in BC patients. Calibration curves and DCA showed good discriminative ability and potential clinical net benefit of this nomogram. These results strongly suggest the applicability of our nomogram.

The immune response exerts a dominant role in cancer progression and could serve as a tumor therapeutic target [49]. TMB is a somatic biomarker proposed to predict response to immunotherapies in cancer [27]. This difference was not statistically significant (P = 0.077), although considerably high levels of TMB were observed in the high-risk group. Importantly, we noticed a remarkable difference in the survival analysis of TMB between the low- and high-risk groups. These finding indicated that a high TMB is related to better outcome of immunotherapy, which was consistent with the precious studies [50]. We then analyzed the landscape of immune cell infiltration and found that high-risk patients possessed higher levels of M2 macrophages, which was consistent with previous findings whereby high infiltration of M2 macrophages was associated with a worse prognosis in BC [51]. Interestingly, the infiltration of CD8+ T cells exhibited a positive correlation with a risk score, which conflicted with the antitumor effect of these components [52]. Checkpoint blockade immunotherapies have become an essential therapeutic strategy in various malignant tumors [53]. Our results showed that the high-risk group expressed higher levels of many immune checkpoint molecules, from which these patients may produce more clinical responses. In addition, we also noticed that BC patients with a low-risk score had a higher TIDE score than those with a high-risk score, indicating less benefit from immunotherapy. The above results suggest that cuproptosis was related to the immune status, and this signature may help predict the response to immunotherapy in BC.

Urinary bladder instillation chemotherapy is one of the main treatments for bladder cancer [54]. We calculated the IC_50_ of common agents in the low- and high-risk groups and found that patients in the high-risk group were significantly more sensitive to gemcitabine, while cisplatin had higher IC_50_ values in the low-risk group. These findings are partially consistent with previous studies showing that gemcitabine-cisplatin chemotherapy is the standard first-line treatment for advanced bladder cancer [55]. Therefore, the risk score might distinguish more suitable patients to receive appropriate chemotherapies.

Limitations exist for this study. First, this was a retrospective study, which harbors inherent limitations. Second, this prognostic signature was constructed and validated using the public database, multicenter large-scale prospective research is required to evaluate its clinical practicality. Finally, further experiments were required to reveal the biological functions and concrete mechanisms of the CRLs.

In summary, we constructed and validated a prognostic signature composed of 7 CRLs through a series of bioinformatics, demonstrating good accuracy in predicting the survival outcomes of BC patients. Importantly, this prognostic signature might contribute to characterizing the immune status of BC patients and predicting the effect of immunotherapy. Consequently, a comprehensive assessment of CRLs is of great clinical, implications and may provide a significant basis for future studies in BC patients.
